# The Clinical and Genetic Characteristics in Children with Idiopathic Hypogonadotropin Hypogonadism

**DOI:** 10.1155/2022/7973726

**Published:** 2022-09-19

**Authors:** Qiong Zhou, Wenbin Sheng, Suhong Yang, Chaochun Zou

**Affiliations:** ^1^Hangzhou Children's Hospital, Hangzhou 310005, China; ^2^Children's Hospital of Zhejiang University School of Medicine, Hangzhou 310052, China

## Abstract

**Background:**

Idiopathic hypogonadotropin hypogonadism (IHH) is caused by hypothalamic-pituitary-gonadal axis dysfunction. This is divided into Kallmann syndrome which has an impaired sense of smell and hypogonadotropin hypogonadism with normal olfactory (nIHH sense. Approximately 60% of patients are associated with Kallmann syndrome, whereas there are approximately 40% with hypogonadotropin hypogonadism (nIHH). This disease is associated with various variants in genes along with different phenotypic characteristics, and even those gene variations could also lead to the cancer formation in patients. So, current study has been designed to investigate and to better understand the characteristics of various IHH-associated genes and the correlation between IHH genes and phenotype.

**Methods:**

The cohort included 14 children with IHH (6 patients of KS and 8 patients of IHH), including 13 boys and 1 girl. Exclusion criteria are as follows: diagnosis of secondary hypogonadotropin hypogonadism due to tumor, trauma, drugs, or other systemic diseases. Clinical data and genetic results were analyzed.

**Results:**

Almost all male patients showed micropenis (12/13, 92.3%), and few of them had cryptorchidism (5/13, 41.7%). A total of 6 genes, CHD7, PROKR2, ANOS1, FGFR1, SEMA3A, and NDNF, were detected. CHD7 was the most common (11/17, 64.7%), and the main mutation type was missense mutation (14/16, 87.5%). Six reported variants and 10 new variants (5 genes, including entire ANSO1 duplicates) were found. Neonatal variation was detected in 3 patients with IHH. Eight patients inherited the variation from their father, while five patients inherited it from their mother. One patient had both FGFR1 and SEMA3A gene variants, while the other had two different CHD7 gene variants and entire ANSO1 repeats. According to ACMG criteria, 4 variants were pathogenic (P), 2 were possibly pathogenic (LP), and 8 had uncertain significance (US). In patients with P or LP (5/6, 83.3%), we found that extragonadal symptoms were more common.

**Conclusions:**

It was concluded that variations in the studied genes could lead to the IHH. Ten new variants have been reported which may lead to different symptoms of IHH. For CHD7 variants, the rare sequencing variants (RSVs) of P or LP showed commonly associated with CHARGE syndrome. Findings of the current study may help for the better diagnosis and treatment of IHH.

## 1. Introduction

Idiopathic hypogonadotropin hypogonadism (IHH) is due to the hypothalamic gonadotropin-releasing hormone (GnRH) neuron damage. This damage leads to impair secretion, or insufficient action, or decrease in pituitary gonadotropic hormone secretion which results in lack of sex gland function [1]. Its incidence is 1-10/100,000 with a male to female ratio of about 3.6 : 1 [1]. According to the presence of olfactory abnormalities, it can be divided into Kallmann syndrome (KS) and idiopathic hypogonadotropin hypogonadism of normal olfactory (nIHH). IHH has genetic heterogeneity and more than 40 genes have been confirmed to be associated with IHH, which accounts for more than 50% of all patients [2, 3]. In fact, the list of uncommon genes and candidate genes continues to grow [4]. Anosmin-1 gene (ANOS1), also named KAL1, is dominated by X-linked inheritance, and CHD7, FGFR1, FGF8, PROKR2, and SOX10 are dominated by autosomal dominant inheritance. In addition, autosomal recessive FEZF1 and PROK2 are mainly inherited in families with high suspicion of KS. In recent years, IL17RD, SEMA3A, SEMA3E, NDNF, and ANOS1 genes have been found to be closely related to olfactory sense. In addition to hypothalamic gonadotropin-releasing hormone (GnRH) neurons and olfactory damage, several gene mutations have also been found to be associated with other physical abnormalities. ANOS1 may present unilateral renal hypoplasia [5], CHD7 may be associated with tooth development, hearing abnormality, short stature, and intellectual impairment, and FGF8 may be associated with hearing loss and cleft lip and palate, while FGFR1 may be associated with tooth development and cleft lip and palate [5]. Nevertheless, the association between the characteristic IHH phenotype and genotype has not been fully established. The purpose of this study was to better understand the characteristics of IHH genes and the correlation between IHH genes and phenotype, so as to help make better treatment plans for IHH patients and lay the foundation for further research.

## 2. Materials and Methods

### 2.1. Sampling and Study Plans

We reviewed all IHH patients with genetic diagnosis in the Children's Hospital of Zhejiang University School of Medicine and the Hangzhou Children's Hospital from 2017 to 2022. Their family history, clinical features, biochemical indicators including sexual hormone, imaging (bone age, sexual gonad, olfactory bulb, and pituitary), and genetic testing were collected.

Inclusion criteria are as follows: for children > 13 years of age in females or >14 years of age in males: (1) no spontaneous pubertal development or pubertal development arrest, (2) the level of sex hormones showed prepuberty (lower than normal, i.e., male serum androgen level ≤ 1 ng/mL and female serum estrogen level ≤20 pg/mL) [6], (3) there were no space-occupying lesions in imaging of hypothalamus and pituitary region, and (4) chromosome karyotype is normal and for female < 13 years old or male < 14 years old, (1) KS was diagnosed by MRI olfactory bulb, (2) the absence of minipuberty and , low levels of gonadotropins and sex hormones, and (3) genetic tests that may support diagnosis. Exclusion criteria are as follows: diagnosis of secondary hypogonadotropin hypogonadism due to tumor, trauma, drugs, or other systemic diseases.

This study was approved by the Ethics Committee of Children's Hospital of Zhejiang University School of Medicine and Hangzhou Children's Hospital.

### 2.2. Gene Analysis

In general, gonadal panel and whole exon sequencing (WES) were used for detection, and the genes contained were all IHH gene pointed out in the consensus [2]. Through ClinVar (http://www.ncbi.nlm.nih.gov/clinvar), pathogenic mutations were checked. Data interpretation rules were followed as per ACMG guidelines. For the variable names, refer to the rules of HGVS (http://www.hgvs.org/mutnomen/).

### 2.3. Annotation for Variants

Various database searches, general population database search, disease database search, literature search, mutation type specificity analysis, and computational prediction were performed. Allele frequencies in the gnomAD database were used to calculate OR and define RSV (MAF < 0.0001). Computational prediction consists of three parts: pathogenicity prediction in computer tools, alignment conservative analysis, and 3D visualization in 3D modeling software. Six computer tools (SIFT [7], Polyphen-2 [8], PROVEAN [9], Mutation Taster [10], CADD [11], and MetaSVM [12]) are used for predicting the pathogenicity of missense or code shifter, and two (Splice Site Score calculation and SpliceAI [13]) are used for splicing sites. We used Clustal W to align each human gene sequence with homolog of 25 other related species in UniProt database to judge the evolutionary conservation of each amino acid site. The more conserved the site, the more important it is for protein function, which indirectly reveals its pathogenicity. Amino acid changes were visualized using 3D modeling software ChimeraX. Using these spatial models, we compare the nature changes of wild and mutant amino acids and their contact relationships with other amino acids to predict the potential pathogenic effects of specific mutations on proteins.

### 2.4. Statistical Analysis

SPSS version 23 software package was used to check the normal distribution of continuous variables. Normal distribution variables are described by mean ± standard deviation, and nonnormal distribution variables were described by median and quartile distance. Category variables were expressed as percentages.

## 3. Results and Discussion

### 3.1. Clinical Features and Auxiliary Examination

The age of diagnosed children ranged from 2.1 to 18.7 years with a mean age of 12.44 ± 4.98 years. There were 13 males and 1 female. Among the 13 male children, 11 (84.6%) were 7.9 to 18.7 years old, and 6 (46.2%) were ≥14 years old. The female patient was diagnosed at 16.2 years old. There were 6 patients (42.9%) of KS and 8 patients (57.1%) of nIHH. In terms of genital characteristics, among the 13 male children, 12 patients (92.3%) had micropenis or (and) cryptorchidism, including 6 (50.0%) of simple micropenis and 5 (41.7%) of micropenis and cryptorchidism. There was no simple cryptorchidism, but one (nIHH3) had micropenis combined with penis descent and scrotal division. Of the 14 patients, 2 (14.3%) had normal genitalia.

**Figure 1:**
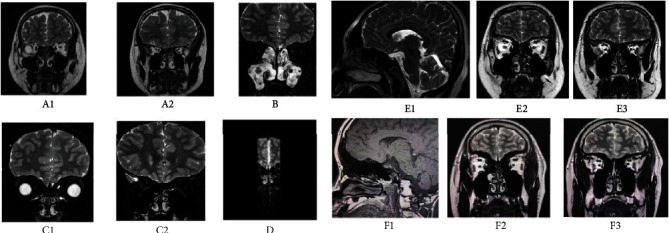
MR abnormalities of olfactory bulb in 6 boys with IHH.

In other clinical manifestations, 4 patients (28.6%) were obese or overweight, of which nIHH1, nIHH5, and nIHH7 were obese and nIHH2 was overweight. Three patients (21.4%), including KS1, KS2, and nIHH5, had short stature. Two patients (14.3%), KS5 and nIHH8, had slurred speech and mental retardation. One (7.1%, KS1) had psychomental abnormalities and gradually developed depression after diagnosis. One (7.1%, KS5) had hearing damage (mainly left ear), male breast development, and hypopigmentation in addition to pronunciation and intelligence defects. One (nIHH1) had a history of nephrotic syndrome at the age of 3 and had received glucocorticoids for 2 years with stable controlling. The only female patient (nIHH8) had a history of ovarian teratoma in addition to slurred speech and mental retardation. nIHH5 is not only short stature and obesity but also diabetes and fatty liver caused by obesity. In the family history, 3 (21.4%) had fathers or mothers with delayed pubertal development, with KS3 and nIHH7 as fathers and nIHH6 as mothers. In laboratory tests, except for one male infant, the luteinizing hormone (LH), follicle-stimulating hormone (FSH), and testosterone (T) measured at 6 months were 0.28 mIU/mL, 0.91 mIU/mL, and 0.2 ng/mL, respectively. The levels of LH, FSH, and T in 1 infant and 3 school-age boys were all lower than the normal level in this age group. T of 8 male children ≥ 12 years old were all lower than 0.5 ng/mL, and their LH and FSH levels were normal or lower than normal [14, 15]. In one female child, LH, FSH and estradiol (E2) were far below the normal low limit [14, 15]. Five of the 13 boys underwent human chorionic gonadotropin (HCG) stimulation test to assess testicular function, of which only one was normal (testosterone > 1 ng/mL), and the other four did not reach 1 ng/mL, nor increased by more than 3 times compared with the baseline value. Five patients underwent gonadotropin-releasing hormone (GnRH) stimulation test. The statistics of the post-LH and -FSH levels ranged from 0.44 mIU/mL to 6.16 mIU/mL and 1.11 mIU/mL to 6.3 mIU/mL, respectively. B ultrasound of testis in 13 boys showed that testis was significantly smaller than normal range. All of the above can be seen in Tables 1 and 2. In the imaging examination, the girl's uterus and ovary B ultrasound indicated that she was in the state of puberty. The pituitary MR of 14 patients showed no space occupying or organic lesions, MRI examination of olfactory bulb was completed in 10 boys, and absence or dysplasia of olfactory bulb, olfactory groove, and olfactory bundle was found in 6 boys (Figure 1).

### 3.2. Molecular Genetic Analysis

A total of 7 genes associated with IHH were identified in 14 patients, including 10 of CHD7, one of NDNF, one of ANOS1, one of FGFR1, one of SEMA3A, one of FGF8, and one of PROKR2, respectively. There were 16 variants in 14 patients. Copy number variation of ANOS1 was found in one patient. The remains were all point mutations, including 2 splicing site mutations of CHD7 gene (KS1 and KS4), one coding mutation (insertion) of PROKR2 gene (nIHH7), and 12 missense mutations. nIHH1 has two missense mutations of CHD7 and ANOS1 repeat. nIHH5 had a missense mutation in FGFR1 and SEMA3A, respectively. Neonatal variation was found in 2 patients with IHH; 9 of the patients inherited the variation from their father and 5 from their mother, both of whose parents carried the heterozygous variation. There were 2 patients with c.1565G>T(p.G522V) variant.

Among the 15 point mutations, there were 9 novel variants including 2 splice site mutations and 1 frameshift mutation. The amino acid sites of 5 CHD7 variants were retrieved from ExAC database: c.409T>G(p.S137A), c.749G>A(p.R250H), c.1565G>T(p.G522V), c.59G>A(p.G20D), and c.2182G>A (p.D728N). However, in each gene variation database, CHD7 retrieved c.2182G>A(p.D728N), NDNF, FGFR1, FGF8, and SEMA3A but did not retrieve the mutations in this cohort. CHD7 c.1565G>T(p.G522V) in ClinVar database was reported in CHARGE syndrome, primary ovarian insufficiency, KS, and other diseases, and in MASTERMIND database notes 2007-2022, this variant was reported in as many as 10 studies. The mutation frequency was significantly higher than that of other mutations and was classified as hot spot mutation and currently classified as benign or likely benign. CHD7 c.409T>G(p.S137A) in the ClinVar database was also reported to be benign or likely benign, with no specific disease description. Other studies reported c.2182G>A(p.D728N) of CHD7 in IHH or CHARGE syndrome [16–18], c.1369A>G(p.T457A) of SEMA3A [19], and c.749G>A(p.R250H) of CHD7 in abnormal sexual development [20]. At the same time, no focal duplication of ANOS1 gene has been reported, and only multiple abnormal patients with multiple gene duplication have been reported [21]. After ClinGen and ClinVar data retrieval, at present, only prompt ANOS1 has sufficient evidence for haploinsufficiency, but no evidence for triplosensitivity; hence, it is currently considered to be uncertain significance.

**Figure 2:**
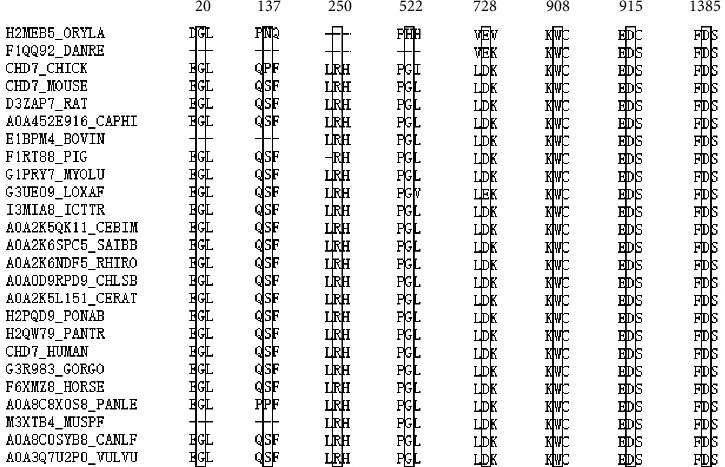
Sequence alignment of CHD7 protein from 25 different species.

**Figure 3:**
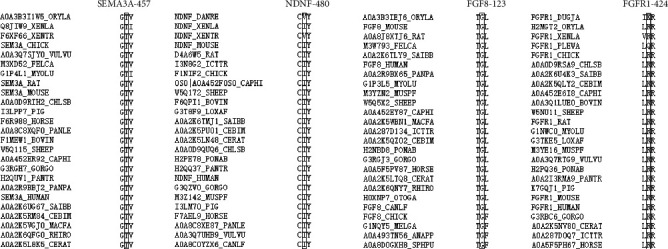
Sequence alignment of four other proteins from 25 different species.

**Figure 4:**
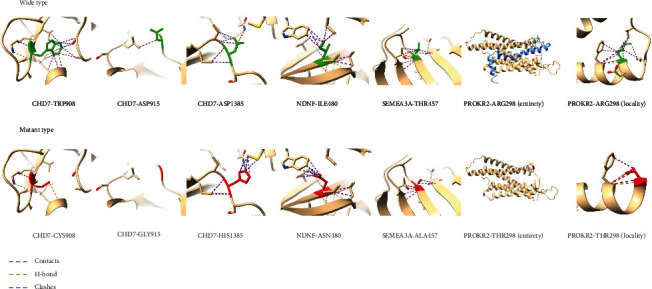
3D structural modeling of wild and mutant proteins.

We calculated pathogenicity predictions for all variants except copy number duplicates. First, the prediction results of c.409T>G(p.S137A) and c.1565G>T(p.G522V) of CHD7 in the three missense mutation software were consistent with the benign or likely benign conclusion suggested in the database (none of which was defined as pathogenicity). The remaining 10 missense mutations and 7 variants were predicted to have obvious pathogenicity. The c.59G>A(p.G20D) and c.2182G>A(p.D728N) amino acid sites of CHD were predicted to be pathogenic in only one of the three software. The R424H variant of FGFR1 was positive for all three predictors, but its predictive value was close to normal (Table 3). Then, 10 missense mutations (except c.409T>G(p.S137A) and c.1565G>T(p.G522V) of CHD7) were calculated again with three software of predictable coding and noncoding regions. The pathogenicity of the two splicing sites was predicted. Results suggested pathogenicity in all (at least 2 out of 3) (Table 4). Second, using all 12 missense mutations as subjects, the results showed that in c.59G>A(p.G20D), c.749G>A(p.R250H), c.2724G>T(p.W908C), c.2744A>G(p.D915G), and c.4153G>C(p.D1385H) of CHD7, c.1369A>G(p.T457A) of SEMA3A, and c.368G>A(p.G123E) of FGF8, wild-type residues at these seven specific sites were highly conserved in 25 different species (Figures 2 and 3). Third, since 3D models of Trp908Cys, Asp915Gly, and Asp1385His for CHD7, Thr457Ala for SEMA3A, Ile480Asn for NDNF, and Arg298Thrfs∗2 for PROKR2 (manufactured by Swiss Model) are available, we can see macroscopic changes in protein structure as well as the direct effects of individual amino acid changes. For all six variants, the residue size, charge, and hydrophobicity varied at specific sites. In addition, the Arg298Thrfs∗2 of PROKR2, due to frameshift variation, leads to the premature termination of protein synthesis and the disappearance of long sequences of amino acids on the structure. In CHD7, for Trp908Cys, 16 contacts disappeared, forming a new hydrogen bond (H-bond). For Asp915Gly, it changed from acidic amino acid to nonpolar hydrophobic amino acid. One old contact broke. For Asp1385His, from acidic amino acid to alkaline amino acid, 4 old contacts and 2 H-bonds broke; meanwhile, 6 new contacts and 1 clashes formed. For NDNF Ile480Asn, nonpolar hydrophobic amino acids became acidic, changing from 6 old contacts to 8 new contacts, and 1 clash formed. For Thr457Ala of SEMA3A, the polar neutral amino acid becomes nonpolar hydrophobic, and 2 new contacts and 1 H-bonded formed. For Arg298Thrfs∗2 of PROKR2, in addition to the disappearance of amino acid sequence, there was also a great change at the termination codon, from alkaline amino acid to polar neutral amino acid, 7 contacts lost, and 1 new H-bond formed (Figure 4). According to the above findings, combined with classification according to the guidelines of ACMG, 4/16 (25%) variants were classified as pathogenic, 2/16 (12.5%) as likely pathogenic, and 8/16 (50%) as uncertain (Table 4).

IHH is an inherited and clinically heterogeneous disease. Different pathogenic genes produce similar clinical phenotypes, and the same pathogenic genes have different clinical characteristics. KS patients are more likely to have abnormal olfactory function, but it is also common for patients with abnormal olfactory bulb to have normal olfactory function. In this study cohort, most male patients had reproductive system abnormalities with a high incidence, such as micropenis (92.3%), cryptorchidism (41.7%), penis retraction (7.7%), and scrotal division (7.7%), which were consistent with literature reports [16–19]. These findings suggest the possibility and necessity of early diagnosis of IHH. Abnormal male gonadal development may indicate defects in the HPG axis during embryonic development [22]. Therefore, for male children with the above abnormalities found in the neonatal period, based on existing and ongoing studies on the reference range of sex hormones and other endocrine hormones in children of all ages [14, 15], the sex hormone profile of 4-8 weeks can be used to diagnose IHH [23–25]. There were very few female patients, only 1 case, and indeed no abnormal manifestations of secondary sexual characteristics. This is similar to previous reports [26, 27]. After entering puberty, the female patient delayed menarche and was found to have ovarian teratoma, which has since been diagnosed. This woman also had a number of nonreproductive abnormalities, and other IHH patients in the cohort also had various types of extragonadal abnormalities, including overweight or obesity, short stature, hearing impairment, mental retardation, pronunciation impairment, hypopigmentation, and mental abnormalities. This laid a foundation for us to study the relationship between IHH gene type and clinical phenotype.

The sex hormone levels of the IHH group were generally low in the study. IHH is caused by decreased GnRH secretion in the hypothalamus or by dysregulation of its receptor. Testis function is normally normal. In this study, 4/5 male children had poor response to the standard HCG test. Unfortunately, no prolonged test was conducted to confirm this. The GnRH test does not determine whether gonadotropin deficiency is caused by hypothalamus or pituitary gland and may be negative in patients with hypothalamic gonadotropin deficiency and positive in some patients with pituitary deficiency. In this cohort, 3/6 children had LH peak value < 4 mIU/mL, and some studies believed that gonadotropin-releasing hormone stimulation test was suggestive for the differential diagnosis of IHH and CDGP, and LH peak value < 4 mIU/mL was meaningful for the diagnosis of IHH [28]. However, there were also 3/6 patients with LH peak value > 4 mIU/mL, of which 2/6 patients > 5 mIU/mL. KS5 and nIHH8 genitalia showed no obvious abnormalities and entered the Tanner stage 2. This may be because gonadotropin pulsating patterns in IHH patients have a fairly wide range of abnormal developmental patterns, from the complete absence of GnRH-induced LH impulses to sleep-induced GnRH release, indistinguished from early adolescence [29–31]. This broad spectrum of neuroendocrine activity explains the various reproductive phenotypes observed in patients with IHH [32]. Olfactory function examination and MRI examination of olfactory organs in 286 patients with IHH and 2183 normal controls found that IHH patients' self-evaluation of olfactory far underestimated the proportion of true olfactory defects. The results of olfactory function examination showed that all the patients who complained of olfactory abnormalities had olfactory abnormalities, so the chief complaint of anosmia was reliable. In this study, olfactory function was mainly evaluated by children and parents during consultation, which may underestimate the proportion of olfactory abnormalities, but it is still reliable for patients with olfactory defects with clear complaints.

**Figure 5:**
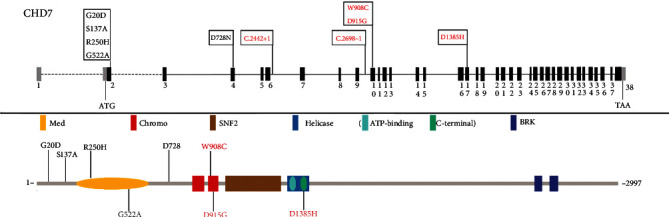
Schematic diagram of CHD7 gene and protein.

The main gene detected in our cohort was CHD7: 11/17 (64.7%). Among the 6 variants assessed as pathogenic or possibly pathogenic, 5 were CHD7 genes (83.3%), which was significantly different from many study cohorts. CHD7 accounted for 4% [33], 8.2% [34], and 26.7% [26, 27] of the detected genes in multiple cohorts. CHD7 is a large nucleoprotein containing two N-terminal chromosomal domains, a central Snf2-like ATPASE and helicase domain, a histone/DNA binding SANT domain, and two C-terminal BRK domains. Our variation distribution in the first half, CHD7 gene and protein area tend to gather at the genetic model of exon 2 and 10 around, protein model on the distribution regularity, no known protein model function domain, and of pathogenic or possibly pathogenic variation is not show the inclination “hot spots” (see Figure 5). To further explore unknown protein regions, we looked them up on the InterPro website and found no conservative areas. This may be hypothetical evidence that rare CHD7 variants in humans may cause various phenotypes of IHH, which is only a milder manifestation of CHARGE syndrome and is also reported to be supported by Kim et al. [35] and Bergman et al. [36]. In their patients with CHARGE syndrome, pathogenic missense mutations mainly occurred in the functional domain aggregation region of CHD7 gene. Clinical features that have been reported that may be associated with CHD7 gene are high palatal arch or cleft palate, dental hypoplasia, auricle dysplasia, perceptual deafness and semicircular canal hypoplasia, short stature, mental retardation, eye defect, or coloboma [35, 37, 38]. These may be monogenic or oligogenic inheritance. Anosmia is not absolutely related to CHD7.

Therefore, both KS and nIHH have CHD7 variants detected in the queue. The presence or absence of anosmia depends on penetrance of the gene, especially in the case of penetrance, especially in the heterozygous state. With the exception of KS3 and nIHH2 (variants classified as benign and possibly benign), 5 of the 8 children with CHD7 variants detected had extragonadal abnormalities (62.5%). Both KS1 (C.2442+1G>A of CHD7) and KS2 (C.2744A>G of CHD7) showed short stature, with olfactory abnormalities and olfactory bulb abnormalities. Both KS5 (C.2724G>T of CHD7) and nIHH8 (C.4153G>C of CHD7) had mental retardation. KS5 found a hearing deficit. These are consistent with known reports. In addition, KS5 and nIHH8 also have pronunciation defects, which may also be related to CHD7 gene, and more sample studies are needed. However, the variants assessed as P or LP (4/5, 80%) were more common to have extragonadal manifestations than the single-gene variants assessed as US (0/2, 0%), which was consistent with the report by Sun et al. [16]. nIHH1 detected 3 gene variants, and on the condition that the pathogenicity of each variant (including the CHD7 variant classified as US) was not clear, the link between gene and phenotype does not allow the possibility of a linear correlation. Similarly, detailed phenotypic analysis of 17 patients reported by Xu et al. [39] showed that 80% (4/5) of patients with P or LP variants showed multiple CHARGE features (mostly extragonadal abnormalities), compared with 8% (1/12) of patients with nonpathogenic (US, B, and LB) variants. The B or LB variants assessed in this group also showed no extragonadal abnormalities (0/2, 0%).However, in Jongmans et al. [40] and Bergman et al. [41], there was no association between genotype and phenotype in CHARGE syndrome patients. However, the sample size of patients with P and LP variants was small, so US variants need to be further confirmed by functional tests or/and reclassified with additional evidence. Therefore, variations in specific clinical manifestations that may provide information related to genetic types need to be carefully interpreted.

Another variant evaluated as P in our cohort was C. 891_892insA of PROKR2, which not only resulted in a change in a single amino acid but also resulted in the termination of all nucleotide encoding after the mutation site due to frameshift mutation and the disappearance of amino acid sequence. Prokineticin-2 (PROK2) is a protein that plays an important role in olfactory nerve development. Human regulation of GnRH neurons and physiology requires its receptor PROKR2. PROKR2 was first reported in 2006 to be associated with syndromic hypogonadotropin with/without anosmia [42]. RSV in PROKR2 is always heterozygous, as reported by this patient (nIHH7) and others [38]. Combined with the fact that the father of the child has a history of CDGP, the child may have a reversal of reproductive defects. Some heterozygous variations in PROKR2 may act in a dominant inhibitory manner [43]. PROKR2-associated hypogonadotropin hypogonadism has been hypothesized to be caused by the interaction of other gene products, since the overexpression of the variant allele does not inhibit the function of the coexpressed wild-type protein [44]. Larger queues are needed to validate the results. The extragonadal manifestations of the children were only obesity, without synkinesia, and other manifestations, but there were few nonreproductive manifestations similar to those reported previously [38]. SEMA3A is a key signaling protein for axon development and plays an important role in many physiological processes. It is involved in axon rejection, dendrite branching, synaptic formation, and neuronal migration by binding NRP1, NRP2, and PLXNA complex receptors. nIHH5 has both SEMA3A c.1369A>G and FGFR1 c.1271G>A variants. Here, the focus is on the SEMA3A variant recently reported by Dai et al. [19], which is not a new variant. This study provides strong evidence to support its pathogenic role in patients with nIHH. The study identified families with the mutation in 196 patients with IHH. Interestingly, the child carried 5 variants of 5 genes (including this variant), the mother carried 4 variants of the other 4 genes, and both the father and one sister carried only c.1369A>G (SEMA3A).

The other sister had one of the five genes and the corresponding variant (not c.1369A>G of SEMA3A), and only the child had IHH manifestations, while the other four had normal phenotypes. At the same time, the researchers completed functional tests of the variant. The results suggest that the SEMA3A variant (c.1369A>G(p.T457A)) leads to defects in FAK phosphorylation and GN11 cell migration and supports its pathogenic role in nIHH patients. However, as it is a single experimental evidence, according to ACMG genetic classification, this experiment has not been reported and verified so far, so it cannot be classified as reproducible and confirmed as stable and effective. Therefore, it cannot be applied to PS3 evidence and can only be evaluated as PP5 evidence. Moreover, PP6 was temporarily classified as US because the evidence came from the conserved judgment sites of homologous alignment of other species and the prediction of protein model software of computer. We need to validate the changes in protein function caused by this variant and further strengthen the experimental evidence to support the hypothesis of its pathogenicity. The FGFR1 protein is a member of the receptor tyrosine kinase (RTK) superfamily. FGFR1 signaling has been shown to play critical roles in the development of the olfactory system, as well as normal GnRH neuronal migration, differentiation, and survival within the hypothalamus. The pathogenesis of nIHH5 follows the oligogenic pattern of disease development, suggesting that these mutations act synergistically to bring about the IHH phenotype. The nongonadal abnormalities in this child are short stature, obesity, and obesity-related complications, which are not synkinesia, cleft lip, and/or palate; hypoplasia of teeth was mentioned in the literature. Digit malformations [6, 38, 45] and others require a summary of more samples. Another possible oligogenic genetic pattern is nIHH1, which has both c.749G>A and c.1565G>T of CHD7 and whole repeats of ANOS1. Having looked at CHD7 in detail, let us expand on ANOS1. ANOS1, also known as Kallmann syndrome 1 (KAL1) gene, is one of the most common genes involved in IHH and is responsible for the X-linked recessive form of KS. ANOS1 is located on chromosome Xp22.31 and consists of 14 exons encoding an extracellular cell adhesion protein anosmin-1 with 680 amino acids, which is essential for olfactory guidance and migration of olfactory and GnRH neurons from the nasal cavity to their final destination [46]. Because the deletion and variation of this gene has clear pathogenic evidence, it is highly correlated with anosmia or hypoxia, digital synkinesia, high-arched palate, unilateral renal agenesis, and other clinical phenotypes [5]. However, the child in this case only had the duplication of this gene, and in addition to sexual dysplasia, he only had a 2-year history of nephropathy without renal structural abnormalities. At present, there is no clear support for the variation of these genes to cause the IHH phenotype of the child. The last gene to discuss is NDNF. NDNF is a secreted neurotrophic factor that promotes neuronal migration, growth, and survival, as well as the growth of neural processes. A recent study demonstrated statistical enrichment of PTV in NDNF, which encodes glycosylated disulfide proteins in the FN3 domain, by studying 240 IHH-independent precursor bands [47]. The enrichment of PTV in NDNF suggests that deletions in NDNF may explain some patients of IHH. NDNF is expressed in the nasal region after formation of the olfactory placode in mice and humans [47]. The positive effects of recombinant NDNF on GnRH neuronal migration in vitro and the migration defects of GnRH neurons in zebrafish injected with z-ndnf MO and ndnf-null mice provide strong evidence for the role of NDNF in GnRH neuronal migration [47]. Even though c.1439T>A (NDNF) mutation of KS6 is currently classified as US, we should keep an eye on it and complete functional tests to supplement more clinical data of patients associated with this variant.

We believe that more support will be provided in the future.

## 4. Conclusion

Abnormalities in CHD7, PROKR2, ANOS1, FGFR1, SEMA3A, or NDNF genes can lead to IHH, with or without extragenital manifestations. IHH should be highly suspected in males with small penises and/or cryptorchidism. New 6 reported variants and 10 new variants (5 genes, including entire duplicates of ANSO1) were identified in IHH with different symptoms. A small proportion of patients may be affected by oligogenic inheritance. For CHD7 variants, the RSV of P or LP is more commonly associated with CHARGE syndrome. These findings provide more references and suggestions for the diagnosis and research of IHH.

## Figures and Tables

**Table 1:** Clinical characteristics of 6 patients with KS.

No.	Gender	Diagnosis age (y)	Puberty	Height (cm)	Micropenis/cryptorchidism	Specific disease history/associated phenotypes	Family history	TV (mL)	HCG test: basal/post	LHRH test: basal/post		Olfaction/OB on MRI
									T (ng/mL)	LH (mIU/mL)	FSH (mIU/mL)	
KS1	M	13.0	Absent	145.0	+/+	Depressive disorder, short stature		L0.5, R0.4	<0.02/-	<0.1/-	0.4/-	Hyposmia/abnormal
KS2	M	15.6	Absent	152.5	+/-	Short stature		L0.3, R0.5	0.40/0.86	<0.07/1.77	0.61/5.34	Hyposmia/abnormal
KS3	M	13.2	Absent	150.0	+/+		CDGP (father), younger bother with same mutation	L0.95, R0.5	0/0.26	<0.07/-	3.62/-	Normal/abnormal
KS4	M	15.7	Absent	164.8	+/+			L0.53, R0.76	0.13/-	<0.07/6.16	1.92/5.22	Normal/abnormal
KS5	M	18.7	Partial^∗^	170.0	-/-	Left ear hearing impairment, inarticulate, gynecomastia, depigmentation, intellectual defect		L2.5, R3.0	0.44/-	1.3/-	2.6/-	Hyposmia/abnormal
KS6	M	11.7	Absent	141.0	+/-			L0.17, R0.17	0.13/-	0.07/-	1.06/-	Hyposmia/abnormal

^∗^His testicles may have developed in Tanner stage 2 by the age of 15, but there was no progress for the next 3 years. TV, . CDGP: constitutionally delayed growth and development.

**Table 2:** Clinical characteristics of 8 patients with nIHH.

No.	Gender	Diagnosis age (years)	Puberty	Height (cm)	Micropenis/cryptorchidism	Specific disease history/associated phenotypes	Family history	TV (mL)	HCG test: basal/post	LHRH test: basal/post		Olfaction/OB on MRI
									T (ng/mL)	LH (mIU/mL)	FSH (mIU/mL)	
nIHH1	M	7.9	Absent	132.0	+/-	Nephrotic syndrome (2 years of cortisol therapy since age 3), obesity		NA	0.23/-	<0.07/-	0.82/-	Normal/normal
nIHH2	M	10.1	Absent	143.0	+/-	Overweight		NA	0.11/1.47	0.39/-	3.56/-	Normal/normal
nIHH3^a^	M	2.4	NA	94.0	+/-	Congenital penis curvature, scrotal division		NA	0.2/-	0.28/-	0.91/-	NA/normal
nIHH4	M	2.1	NA	91.0	+/+			NA	0.12/-	0.04/-	0.55/-	Normal/NA
nIHH5	M	16.7	Absent	140.0	+/+	Short stature, obesity, diabetes, fatty liver		L0.36,R0.63	0.21/0.38	0.3/1.91	1.23/4.8	Normal/normal
nIHH6	M	14.4	Absent	156.5	+/-		CDGP (mother)	L0.54, R0.6	0.1/0.62	0.31/6.53	0.59/6.3	Normal/NA
nIHH7	M	16.5	Absent	165.7	+/-	Obesity	CDGP (father)	L2.0, R2.5	0.17/0.29	0.2/4.4	1.7/4.6	Normal/NA
nIHH8	F	16.2	Partial^b^	158.5		Ovarian cystic teratoma, inarticulate, intellectual defect			E2:14.9 pg/mL	<0.1/0.44	0.31/1.11	Normal/NA

a: He was only tested for sex hormones when he was six months old. b :She had Tanner stage 2 breast development at 13 years of age and it did not progress for the next 3 years NA, not available. CDGP: Constitutionally delayed growth and development.

**Table 3:** Genetic analysis of 14 children with IHH.

Case	Gene	Variant	Novel	Amino acid	Pathogenicity^b^	Inheritance	Source of variant	MAF (%)	Polyphen-2	SIFT	PROVEAN
KS1	CHD7	c.2442+1G>A	Yes	/	Likely pathogenic	AD, Het	De novo	No	/	/	/
KS2	CHD7	c.2744A>G	Yes	p.D915G	Uncertain	AD, Het	Paternal	No	0.991	0.001	5.7
KS3	CHD7	c.409T>G	No	p.S137A	Likely benign	AD, Het	Paternal	0.020	0.002	0.19	0.62
KS4	CHD7	c.2698-1G>T	Yes	/	Pathogenic	AD, Het	Paternal	No	/	/	/
KS5	CHD7	c.2724G>T	Yes	p.W908C	Uncertain	AD, Het	Maternal	No	1	0.00	12.43
KS6	NDNF	c.1439T>A	Yes	p.I480N	Uncertain	AD, Het	Paternal	No	0.963	0.002	5.23
nIHH1	CHD7	c.749G>A	No	p.R250H	Uncertain	AD, Het	Paternal	0.006471	0.999	0.011	0.76
		c.1565G>T	No	p.G522V	Likely benign	AD, Het	Paternal	0.619	0.099	0.05	0.47
	ANOS1^a^	GRCh38/hg38:chrX:(8528874-8732137)dup			Uncertain	XLR	De novo	No
nIHH2	CHD7	c.1565G>T	No	p.G522V	Likely benign	AD, Het	Maternal	0.619	0.099	0.05	0.47
nIHH3	CHD7	c.59G>A	No	p.G20D	Uncertain	AD, Het	Maternal	0.001057	0.916	0.285	0.44
nIHH4	CHD7	c.2182G>A	No	p.D728N	Uncertain	AD, Het	Paternal	0.01164	0.155	0.011	2.34
nIHH5	FGFR1	c.1271G>A	Yes	p.R424H	Uncertain	AD, Het	Paternal	No	0.060	0.041	2.51
	SEMA3A	c.1369A>G	No	p.T457A	Uncertain	AD, Het	Maternal	No	0.789	0.005	3.58
nIHH6	FGF8	c.368G>A	Yes	p.G123E	Uncertain	AD, Het	Maternal	No	0.974	0.000	4.99
nIHH7	PROKR2	c.891-892insA	Yes	p.R298Tfs∗2	Likely pathogenic	AD, Het	Paternal	No	/	/	/
nIHH8	CHD7	c.4153G>C	Yes	p.D1385H	Uncertain	AD, Het	De novo	No	1	0.000	6.71

a: represents copy number variation, and represents variant if no hint is given. Uncertain is recorded after checking two databases; b: records after checking according to hospital laboratory report and multiple databases. SIFT score: Less than 0.05 is expected to be Deleterious, greater than or equal to 0.05 is expected to be Tolerated. Polyphen-2 score: If the score is between 0.909 and 1, it is Probably damaging;Scores between 0.447 and 0.908 are "potentially Damaging", while 0 and 0.447 are Benign. PROVEAN score: Less than -2.5 is expected to be Deleterious, more than -2.5 is expected to be Neutral AD: autosomal dominant,Het: heterozygous, XLR: X-linked recessive.

**Table 4:** Further genetic pathogenicity analysis of 12 children with IHH (excluding benign or likely benign variants).

Case	Gene	Variant	Amino acid	Source of variant	Mutation taster	CADD_raw	CADD_phred	MetaSVM_score	Classification (ACMG)
KS1^∗^	CHD7	c.2442+1G>A	/	De novo	1	/	/	/	P (PVS1, PS2, PM2, PP3, PP4)
KS2	CHD7	c.2744A>G	p.D915G	Paternal	0.999	4.436	32	0.31	LP (PM1, PM2, PP3, PP4)
KS4^∗^	CHD7	c.2698-1G>T	/	Paternal	1	/	/	/	P (PVS1, PM2, PP3)
KS5	CHD7	c.2724G>T	p.W908C	Maternal	1	4.566	32	1.068	LP (PM1, PM2, PP3, PP4)
KS6	NDNF	c.1439T>A	p.I480N	Paternal	0.999	4.094	28.5	0.508	US (PM2, PP2, PP3)
nHH1	CHD7	c.749G>A	p.R250H	Paternal	0.999	3.505	25	0.08	US (PP3, PP4)
nHH3	CHD7	c.59G>A	p.G20D	Maternal	0.986	2.865	23.3	1.045	US (PP3, PP4)
nHH4	CHD7	c.2182G>A	p.D728N	Paternal	0.999	2.819	23.2	0.488	US (PP3, PP4)
nHH5	FGFR1	c.1271G>A	p.R424H	Paternal	0.999	3.489	24.9	0.062	US (PM2, PP3, PP4)
	SEMA3A	c.1369A>G	p.T457A	Maternal	0.999	3.672	25.6	1.111	US (PM2, PP2, PP3, PP5, PP6)
nHH6	FGF8	c.368G>A	p.G123E	Maternal	0.999	3.812	26.3	1.017	US (PM2, PP3, PP6)
nHH7	PROKR2	c.891_892insA	p.R298Tfs∗2	Paternal	/	/	/	/	P (PVS1, PM2, PM4, PP4)
nHH8	CHD7	c.4153G>C	p.D1385H	De novo	0.999	4.305	31	1.004	P (PS2, PM1, PM2, PP3, PP4)

^∗^: Splice Site Score Calculation and SpliceAI for Splicing Sites: Test positive. The 12 variants predicted by MutationTaster are all classified as "pathogenic". CADD_raw is the initial score, and CADD_phred is the converted score. The higher the score, the greater the harmful effect.The CADD_phred score is recommended to be greater than 15. MetaSVM fractional cut value is 0.0 (higher score indicates greater harmful effects). ACMG: American College of Medical Genetics Laboratory Practice Committee Working Group, Described as P:pathogenic; LP:likely pathogenic; US: uncertain significance. B: benign;LB: likely benign. PVS: pathogenic very strong, PS: pathogenic strong, PM: pathogenic moderate,PP: pathogenic supporting.

## Data Availability

The data supported the research are included in the article.

## Abbreviations

IHH: Idiopathic hypogonadotropin hypogonadism
nIHH: Hypogonadotropin hypogonadism with a normal sense of smell
KS: Kallmann syndrome
ACMG: American College of Medical Genetics Laboratory Practice Committee Working Group
WES: Whole exon sequencing
P: Pathogenic
LP: Likely pathogenic
US: Uncertain significance
B: Benign
LB: Likely benign
RSV: Rare sequencing variant
MAF: Maximum allele frequency
LH: Luteinizing hormone
FSH: Follicle-stimulating hormone
T: Testosterone
H-bod: Hydrogen bond
GnRH: Gonadotropin-releasing hormone
CDGP: Constitutionally delayed growth and development.
